# Author’s Reply: Refining Open-World Game and Nostalgic Film Interventions for Broader and More Reliable Therapeutic Impact

**DOI:** 10.2196/83239

**Published:** 2025-09-30

**Authors:** Andreas Benedikt Eisingerich, Annisa Arigayota, Barbara Duffek, Congcong Hou

**Affiliations:** 1 Imperial College Business School, Imperial College London, South Kensington Campus, London, SW7 2AZ, United Kingdom, 44 207594 ext 9763; 2 Robinson College of Business, Georgia State University, Atlanta, GA, United States; 3 Kyushu Sangyo University, Fukuoka, Japan

**Keywords:** open-world games, nostalgia, lab experimental study, postgraduate students, My Neighbor Totoro, Kiki’s Delivery Service, Nintendo

We greatly welcome and thank Sun and Meng for their thoughtful comments in their letter to the editor [1] in response to our research [2]. We are grateful for the insightful points shared on how work on creative media, such as video games and films, may help challenge stereotypes about gaming and engaging with storytelling to potentially open doors accessible and innovative tools for well-being. Open-world games may have the potential to enhance young people’s mental well-being [3], whilst social media and other forms of internet usage may lead to stress and anxiety [4,5]. We respond to the kind and insightful points shared [2] to further help bridge creative media and mental health science.

First, we concur regarding the stated importance to examine the observed effects in our study [1], using participants who may not already be predisposed to enjoy the stimuli such as graduate students. As we note in our article, we recruited participants by telling them that they had a chance to take part in a university study on daily activities and well-being. Thus, the danger of people who already like watching Studio Ghibli films and enjoy playing The Legend of Zelda: Breath of the Wild to self-select to take part in the study is low. We also controlled for study participants’ familiarity with and enjoyment of the creative art content. That said, we fully agree that that future research should study the impact of creative media on well-being using a larger, broader and more varied sample in terms of different study participant demographics (age, cultural background, etc).

Second, we concur that due to the intervention’s brevity (ie, 30 min of video gameplay and roughly 7 minutes of film viewing), only a snapshot of the possible psychological effect may be captured and may constitute a conservative test. This is an excellent point and we encourage future research to explore the impact of long-term intervention as well as varied intervention formats (eg, multi-session, spaced over several days and weeks and possibly even years) on long-term well-being gains. Additional work is needed to help examine the long-term effects of playing open-world games such as The Legend of Zelda: Breath of the Wild and watching Studio Ghibli classics such as My Neighbor Totor and Kiki’s Delivery Service on mental well-being. In addition, we invite future research to explore the potential effects of games and films other than the ones invested in our study [1] to determine the extent various art forms may lead to benefits in calmness, exploration, enhanced meaning, and sense of purpose in life as well as overall life happiness.

Third, study participants were randomly assigned to the study’s experimental conditions. We agree that the study of how mood, recent stressors, sleep quality (or lack thereof), social interactions, the consumption of one’s favorite beverage (eg, coffee, Red Bull, etc) may interact with the intervention’s effects is richly deserving.

In summary, we thank Meng and Sun for their thoughtful and excellent comments on how future research may help bridge gaming and storytelling with mental health science.
